# Synthesis and Antiproliferative Activities of Conjugates of Paclitaxel and Camptothecin with a Cyclic Cell-Penetrating Peptide

**DOI:** 10.3390/molecules24071427

**Published:** 2019-04-11

**Authors:** Naglaa Salem El-Sayed, Amir Nasrolahi Shirazi, Muhammad Imran Sajid, Shang Eun Park, Keykavous Parang, Rakesh Kumar Tiwari

**Affiliations:** 1 Center for Targeted Drug Delivery, Department of Biomedical and Pharmaceutical Sciences, Chapman University School of Pharmacy, Harry and Diane Rinker Health Science Campus, Irvine, CA 92618, USA; nibrahim@chapman.edu (N.S.E.-S.); ashirazi@ketchum.edu (A.N.S.); sajid@chapman.edu (M.I.S.); park327@mail.chapman.edu (S.E.P.); 2 Cellulose and Paper Department, National Research Center, Dokki 12622, Cairo, Egypt; 3 Department of Pharmaceutical Sciences, College of Pharmacy, Marshall B. Ketchum University, Fullerton, CA 92831, USA; 4 Faculty of Pharmacy, University of Central Punjab, Lahore 54000, Pakistan

**Keywords:** cell-penetrating peptide, conjugate, camptothecin, cytotoxicity, MCF-7, paclitaxel, solubility

## Abstract

Cell-penetrating peptide [WR]_5_ has been previously shown to be an efficient molecular transporter for various hydrophilic and hydrophobic molecules. The peptide was synthesized using Fmoc/tBu solid-phase chemistry, and one arginine was replaced with one lysine to enable the conjugation with the anticancer drugs. Paclitaxel (PTX) was functionalized with an esterification reaction at the C2′ hydroxyl group of PTX with glutaric anhydride and conjugated with the cyclic peptide [W(WR)_4_K(*β*Ala)] in DMF to obtain the peptide-drug conjugate PTX1. Furthermore, camptothecin (CPT) was modified at the C(20)-hydroxyl group through the reaction with triphosgene. Then, it was conjugated with two functionalized cyclic peptides through a formyl linker affording two different conjugates, namely CPT1 and CPT2. All the conjugates showed better water solubility as compared to the parent drug. The cytotoxicity assay of the drugs and their conjugates with the peptides were evaluated in the human breast cancer MCF-7 cell line. PTX inhibited cell proliferation by 39% while the PTX-peptide conjugate inhibited the proliferation by ~18% after 72 h incubation. On the other hand, CPT, CPT1, and CPT2 reduced the cell proliferation by 68%, 39%, and 62%, respectively, in the MCF-7 cell lines at 5 µM concentration after 72 h incubation.

## 1. Introduction

Intensive efforts have been exerted for the development of an intracellular stimuli-responsive drug delivery system that is stable under normal physiological conditions (e.g., in blood circulation) but able to release its payload in the tumor site in a selective and efficient manner [1]. The tumorigenic process and microenvironmental characteristics of the tumor tissue have been extensively studied and used to design and synthesize new targeted molecular therapies [2,3]. More precisely, understanding the tumor microenvironment allows researchers to develop different therapeutic strategies, based on the numerous differences between the tumor microenvironment and the normal tissues [4,5,6]. 

Cell-penetrating peptides (CPPs) are positively-charged short peptides with 5–30 amino acids that are able to penetrate cell membranes and translocate different types of cargos into cells via different mechanisms such as endocytosis [7]. Interestingly, some CPPs have shown attractive therapeutic and biomedical potential due to their biocompatibility and high level of tissue selectivity through binding to specific cell receptors [8,9,10,11,12]. However, due to their peptide-based nature, they have poor in vitro and in vivo stability, which may demote their bioactivity [13]. Synthetic amphipathic CPPs can tolerate the instability of natural CPPs while retaining their ability to permeate the cells and deliver cargos. Furthermore, the physicochemical profile, self-assembly, receptor recognition, and stimuli-responsive properties of amphipathic CPPs can be engineered by selecting the proper amino acid sequences and peptide secondary structures, which are stabilized by hydrophobic–hydrophilic side chain interactions [14]. Rational functionalization of side chains with acid labile, redox sensitive bonds (ester bond and disulfide bridge), and tumor homing peptide motif, e.g., IKVAV, PEG, KGGVG, and RGD, confer remarkable properties to CPPs, such as peptide stability, control over the rate, and site of cargo release [15,16,17].

Several studies have revealed the impact of using CPPs as a molecular transporter for many therapeutic agents, which could bind either through physical loading or via chemical conjugation and could be released by microenvironmental stimuli, such as the pH, redox, irradiation, or enzyme [18,19,20,21,22,23]. 

Mandal et al. [24] reported the synthesis of a series of linear and cyclic CPPs with alternating tryptophan (W) and arginine (R) amino acids. Mandal et al. study revealed that cyclic peptides [WR]_4_ and [WR]_5_ were efficient in translocating the fluorescently-labeled anti-HIV drug (3TC). Furthermore, [WR]_4_ enhanced the cellular uptake for a number of cell-impermeable phosphopeptides, including the fluorescent-labeled F′-GpYLPQTV, F′-NEpYTARQ, F′-AEEEIYGEFEAKKKK, F′-PEpYLGLD, F′-pYVNVQN-NH_2_, and F′-GpYEEI in human leukemia cells (CCRF-CEM) [25]. Furthermore, Shirazi et al. employed the cyclic peptide [W(WR)_4_K] as a molecular transporter for the delivery of doxorubicin (Dox) and curcumin (Cur) to different cancer cell lines by their loading in a physical mixture or via conjugation to the peptide through a hydrolyzable ester bond with glutamate [21,26]. Dox-[W(WR)_4_K] conjugate inhibited the cell proliferation of human leukemia (CCRF-CEM), colorectal carcinoma (HCT-116), ovarian adenocarcinoma (SK-OV-3), and breast carcinoma (MDA-MB-468) cells in a range of 50% to 79%. Flow cytometry evaluation exhibited a 3.6-fold higher uptake of the conjugate versus Dox alone in SK-OV-3 cells. Intracellular hydrolysis of Dox from the conjugate was shown within 72 h incubation. Meanwhile, cellular uptake of Cur by the CCRF-CEM cancer cell line was improved by 5.7- and 4-fold for the physical mixture and the chemical conjugate, respectively, as compared to Cur alone. In the meantime, the antiproliferative activity of Cur in the physical mixture and the chemical conjugate was enhanced by almost 20% and 13%, respectively [21].

Paclitaxel (PTX) is a natural compound isolated from the Pacific Yew tree by Wall and Wani in 1967 [27]. PTX interacts with β-tubulin located inside of the microtubules that induce the microtubules’ polymerization, causing the arrest of the cell cycle at the G2/M phase and inducing apoptosis in cancer cells [28]. Camptothecin (CPT) is a natural product isolated from *Camptotheca acuminate* with anticancer potency. CPT works by inhibiting type I DNA topoisomerase and inducing apoptosis in tumor cells [29]. PTX and CPT have shown broad-spectrum cytotoxic potentiality against ovarian, breast, non-small-cell lung, pancreatic, and brain cancers and Kaposi’s sarcoma [27,30]. Despite the broad-spectrum antitumor activity of PTX and CPT, their clinical applications are hampered by their poor aqueous solubility, limited bioavailability, cellular permeability, lack of tumor selectivity, and serious adverse effects (e.g., myelosuppression and gastrointestinal toxicity) following intravenous administration [30]. Several efforts have been devoted to the development of active formulations of PTX and CPT to improve their water solubility and pharmacokinetic, cell membrane permeability, and tissue selectivity. Nanoparticles, solid lipid nanoparticles, liposomal formulations, hyperbranched block-copolymers, dendrimers, PEGylated polymeric materials, quantum dots, polymeric micelles, and PEGylated poly(L-lysine) (PLL) have shown success, and some of these formulations entered clinical trials [22,30,31,32,33,34,35,36,37,38,39,40].

In continuation of our efforts to design CPP-drug conjugates based on the CPPs containing tryptophan and arginine residues, herein, we report the conjugation of PTX and CPT to the [W(RW)_4_K] peptide and compare the antiproliferative activity of the free drugs with the drug-peptide conjugate in breast cancer cell line MCF-7.

## 2. Results and Discussion

### 2.1. Synthesis of Cyclic Peptide [(WR)_4_K(βAla)] and Its Functionalization

**Scheme 1:**
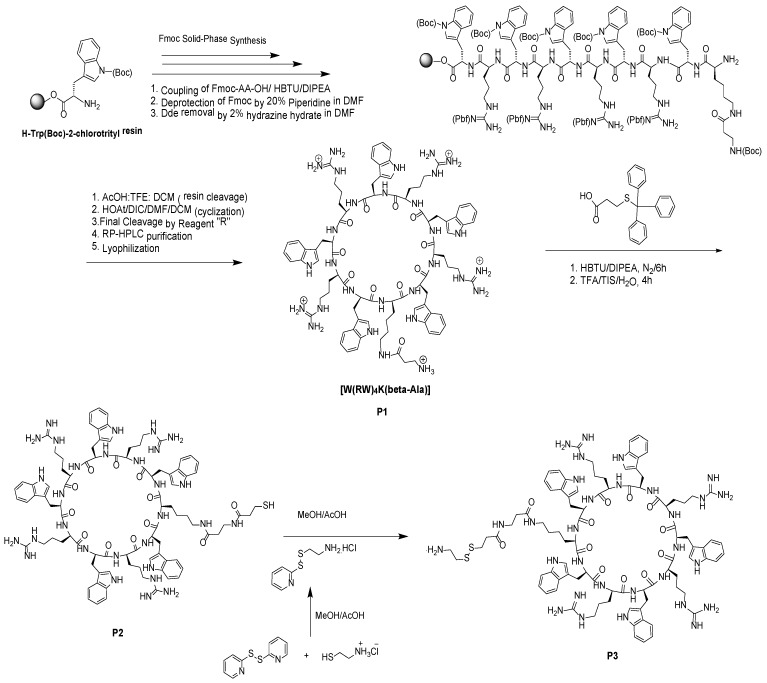
Synthesis of [W(WR)_4_K(*β*Ala)] and its functionalization with tritylthiopropionic acid and 2-(pyridinyldithio)ethaneamine.

**Scheme 2:**
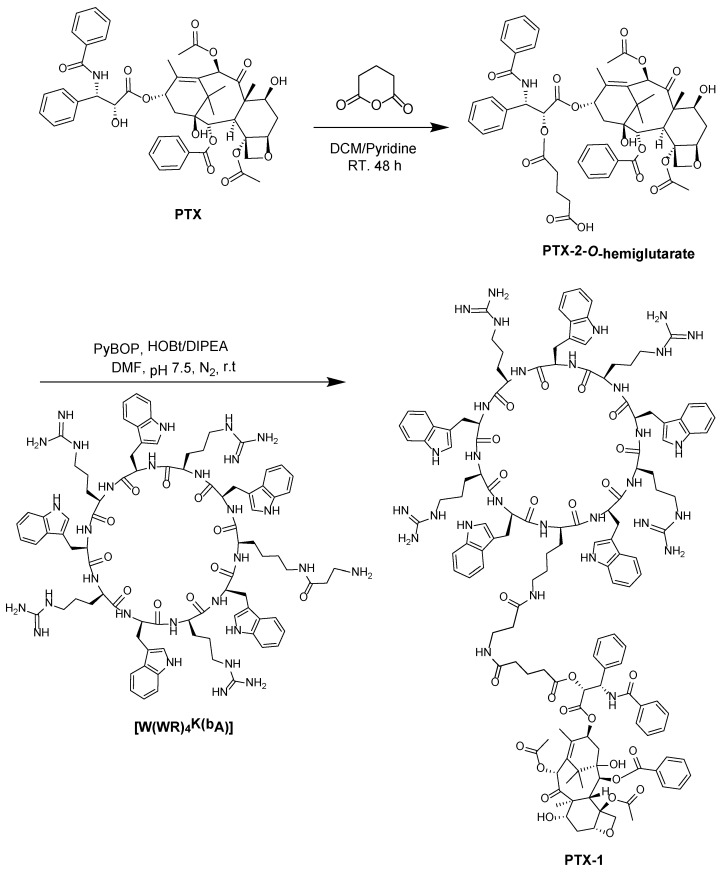
Synthesis of PTX-O-hemiglutarate and its conjugation with [W(WR)_4_K(βAla)].

**Scheme 3:**
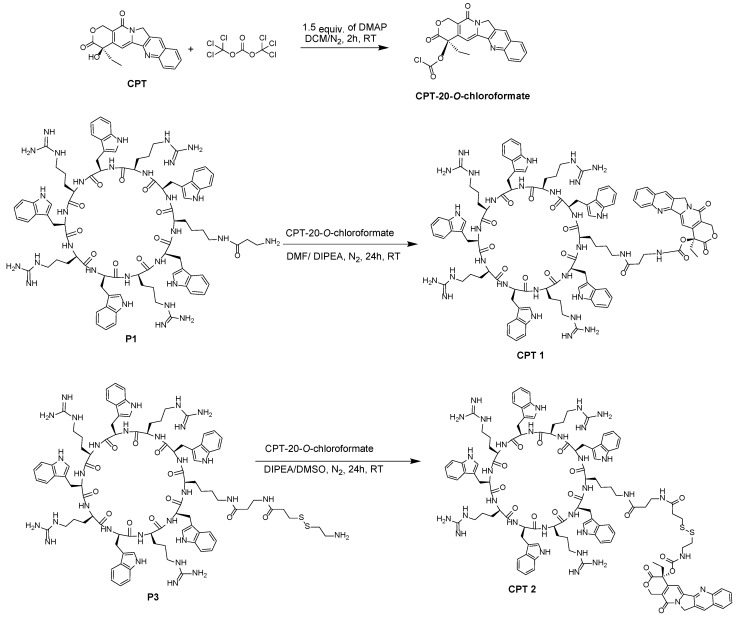
Synthesis of CPT-20-*O*-chloroformate and its conjugation with [W(WR)_4_K(βAla)] and [(WR)_4_W K(*β*Ala-thiopropionyl-*S-S*-ethaneamine)] peptide.

Cyclic peptide [W(WR)_4_K(*β*Ala)] contains alternative l-tryptophan (W) and l-arginine (R) residues and was used in previous studies for the delivery of Cur and Dox to different cancer cell lines [21,26]. [W(WR)_4_K(*β*Ala)] (P1) was prepared through the Fmoc/tBu solid-phase chemistry using appropriate Fmoc-protected amino acids and the orthogonally protected lysine residue with the Dde group [21,26]. The protected cyclic peptide [(W(Boc)R(Pbf))_4_W(Boc)K(βAla-Boc)] was obtained by the cyclization of the side chain-protected linear peptide (HOOC-((W(Boc)R(Pbf))_4_W(Boc)K(βAla-Boc)-NH_2_) in anhydrous DMF/DCM mixture (4:1, *v*/*v*) using HOAt/DIC as activating agents. The completion of cyclization was confirmed by checking the mass of a crude sample taken from the reaction mixture as described in Section 3.2.1. The cleavage of the protecting group using reagent “R” afforded the cyclic peptide P1 with the exact mass of 1754.0300 corresponding to [M + H^+^] (see Supplementary Materials). The thiol functionality was introduced to the cyclic peptide with the coupling of tritylthiopropionic acid in DMF/DIPEA/HBTU. Completion of the reaction was confirmed by mass spectroscopy. The trityl protecting group was cleaved from the thiol group using a mixture of TFA/TIS/H_2_O. MALDI mass spectroscopy revealed the exact mass at 1842.8826 [M − H]^−^ for P2. The last step of peptide functionalization was performed using 2-(pyridinyldithio)ethaneamine which reacted with the cyclic peptide P2. MALDI analysis showed the existence of a single peak at 1917.9098 [M + H]^+^ (see Supplementary Materials) for the final product (P3) as depicted in Scheme 1. P1 was used for the conjugation with both PTX-2-*O*-hemiglutarate (Scheme 2) and CPT-20-*O*-chloroformate (Scheme 3) to afford PTX-1 and CPT1, respectively. P3 was used for conjugation with CPT-20-*O*-chloroformate to afford CPT2 (Scheme 3) as described below.

### 2.2. Coupling Hydrophobic Drugs to [W(WR)_4_K(βAla)]

#### Coupling of PTX to [W(WR)_4_K(βAla)]

PTX is not amenable for coupling unless it is chemically modified. Although PTX has 2′-hydroxyl and 7-hydroxyl groups which are more susceptible to the chemical modifications. However, the 2′-hydroxyl group has superior reactivity than that at the 7-hydroxyl group due to the steric hindrance at this position [38]. Therefore, PTX was functionalized by its reaction with glutaric anhydride, which was reacted with the hydroxyl group at C2′ with the ester linkage. The resulted 2’-*O*-hemiglutarate was conjugated with [W(WR)_4_K(βAla)] (P1) through the amide linkage using PyBOP, HOBt, and DIPEA under N_2_ at room temperature. The conjugate was purified by RP-HPLC and analyzed by MALDI that showed a single peak at 2703.9563 corresponding to [M + H]^+^ (see Supplementary Materials) for the [W(WR)_4_K(*β*Ala-hemiglutarate-2-*O*-PTX)] conjugate (PTX1), as demonstrated in Scheme 2.

Similarly, CPT was conjugated to the [W(WR)_4_K(*β*Ala)] (P1) through an amide bond. Initially, CPT with a C20-chloroformate functional group was prepared by the treatment of CPT with triphosgene in the presence of DMAP. CPT-20-*O*-chloroformate was conjugated with P1 through two different approaches; the first approach was through the pH-labile carbonate bridge, which bound directly to the free *β-*alanyl amino group of the cyclic peptide in DMF using a catalytic amount of DIPEA, as depicted in Scheme 3, to afford carbamate conjugate CPT1. The second approach depends on the reductive-cleavage for the disulfide bridge and hydrolysis of carbonate linkage (the dual response approach) in compound CPT2. Compound P3 was reacted with CPT-20-*O*-chloroformate in the presence of DIPEA to afford CPT2. It was expected that in the second approach, the disulfide bridge in ([W(WR)_4_K(*β*Ala)-*S*-*S*-CPT] would be cleaved via reduction of the disulfide bridge by the intracellular glutathione (GSH). Whereas, the concentration of GSH in the tumor tissue is 4 times higher than that of the normal tissues [40]. Darwish et al. [41] have previously reported the impact of a disulfide bridge in increasing the cytotoxic activity of Dox conjugated to [C(WR)_4_K] when investigated in HEK-293, HT-1080, and SKOV-3 cells as compared to Dox after 72 h incubation. The mechanism affording the thiol-containing CPT followed by the carbonate bond cleavage liberating thiazolidinone and CPT and five-membered thiolactam was demonstrated by Henne et al. and Zhang et al. [39,40].

### 2.3. Biological Activity

Our previous studies revealed that [W(WR)_4_K(*β*Ala)] did not show any significant cytotoxicity up to 10 µM on MDA-MB-468, HCT-116, CCRF-CEM, and SK-OV-3 [21,25,26]. Herein, the antiproliferative activity of conjugate was evaluated in the presence of the human breast adenocarcinoma (MCF-7) cells. Among breast cancer cells, MCF-7 ones were selected to be tested for this assay due to their significant response to microtubule-targeting drugs [42,43]. 

Consequently, the concentration of 5 μM was selected to study the antiproliferation activity of the drug-peptide conjugates. Preliminary cytotoxicity investigation against MCF-7 cell lines showed that both CPT and PTX reduced the cell proliferation of MCF-7 cells at 5 μM by ~68% and 39%, respectively, after 72 h using MTT assay. We have previously shown that the peptide-drug conjugates containing doxorubicin and a similar cyclic peptide containing tryptophan and arginine were cleaved by 99% in the presence of the cancer cells after 72 h of incubation as shown by HPLC analysis [26]. Therefore, 72 h was selected for the incubation of the conjugates in in vitro assays. Further analysis in a shorter time may be beneficial for comparative studies. Although the cells could be incubated with drugs for a longer period, the other elements such as sedimentation of the drug would have complicated the antiproliferative outcomes.

**Figure 1:**
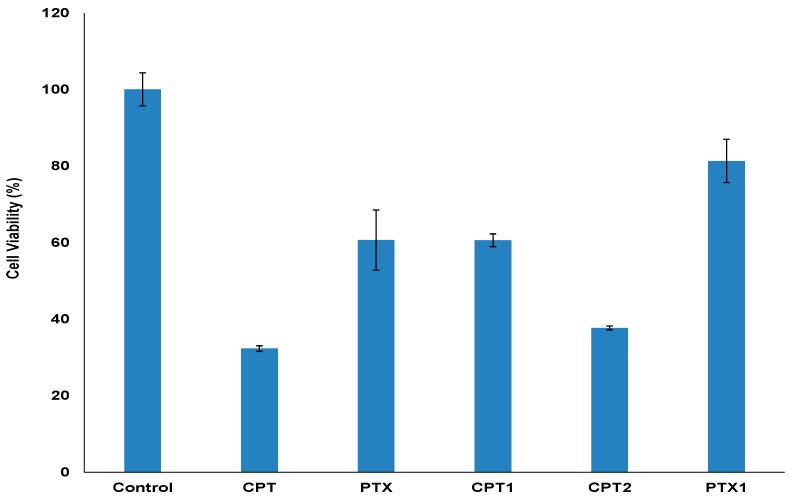
The antiproliferative activity of camptothecin (CPT), paclitaxel (PTX), and their conjugates at (5 µM) concentration in MCF-7 cells after 72 h incubation.

**Figure 2:**
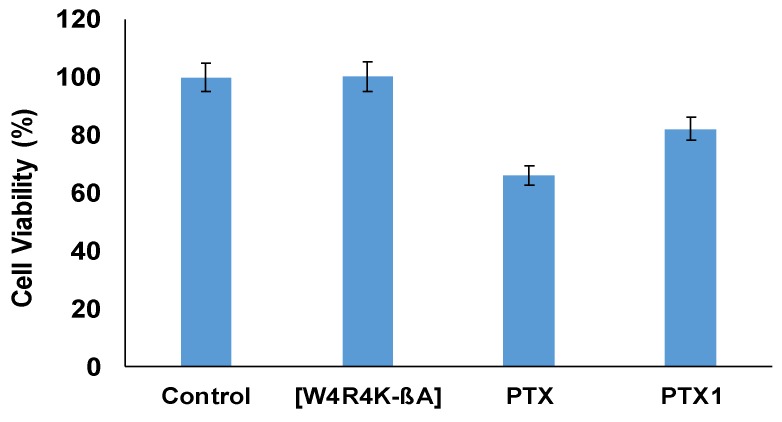
The antiproliferative activity of PTX and PTX1 at (5 µM) concentration in HEK-298 cell line after 72 h incubation.

Antiproliferative results showed that PTX1 inhibited cell proliferation by 18.7%. The anti-proliferative activity of CPT1 was diminished by 1.9-fold as compared to CPT whereas the activity of CPT2 was comparable to CPT, since CPT2 reduced the cell viability to 61% as shown in Figure 1. Since these compounds were peptide-drug conjugates, it was expected that the release of the parent drug would generate the cytotoxic effect. However, PTX1 was not very cytotoxic presumably due to the stability of the conjugate. Among CPT conjugates, CPT2 exhibited higher antiproliferative activity, due to the enhanced release of CPT. We presume that disulfide linker is reduced leading to the release of active CPT from the conjugate. CPT1 and PTX1 conjugates had less activity than the corresponding parent analogs since they act as inactive prodrugs and may behave differently on longer incubation time. The cytotoxicity of PTX and PTX1 was further evaluated in the normal human embryonic kidney cells (HEK-293) at 5 μM which showed reduced cell proliferation by ~34% and 18%, respectively, after 72 h using MTT assay, as shown in Figure 2. The lower cytotoxicity of conjugate PTX1 as compared to PTX in the normal cell lines could be due to slower hydrolysis of PTX from conjugate PTX1.

## 3. Materials and Methods

### 3.1. Materials

All required organic solvents were purchased from Wilkem Scientific (Pawtucket, RI, USA). The Fmoc-protected building block of amino acids, including Fmoc-Trp(Boc)-OH, Fmoc-Arg(Pbf)-OH, Dde-K(Boc)-OH, Boc*-β*Ala-OH, 3-(tritylthio)propionic acid, and H-Trp(Boc)-2-chlorotrityl resin, were purchased from Chem-Impex International Inc. (Wood Dale, IL, USA). Hydroxy-7-azabenzotriazole (HOAT), 1,3-diisopropylcarbodiimide (DIC), *O*-(benzotriazole-1-yl)-*N*,*N*,*N’*,*N’-*tetramethyluroniumhexafluorophosphate (HBTU), benzotriazol-1-yloxytripyrrolidinophosphonium hexafluorophosphate (PyBOP), and hydroxybenzotriazole (HOBt), glutaric anhydride (95%), triphosgene (99%), 4-dimethylaminopyridine (DMAP, 99%), *N*,*N*-diisopropylethylamine (DIPEA), triethylamine (TEA), β-mercaptoethylamine hydrochloride (cystamine hydrochloride), and 2,2′-dithiodipyridine were purchased from Sigma-Aldrich Chemical Co. (Milwaukee, WI, USA). Camptothecin (CPT) was purchased from Santa Cruz Biotech, Inc. (Dallas, TX, USA) and Paclitaxel (PTX) was purchased from Euroasias Group Inc. (Woodbridge, NJ, USA).

### 3.2. Methods

#### 3.2.1. Synthesis of Cyclic Peptide [W(RW)_4_K(*β*Ala)] (P1)

The linear peptide with protected side chains was prepared using Fmoc/tBu chemistry. The peptide sequence was assembled on the H-Trp(Boc)-2-chlorotrityl resin (513 mg, 0.78 mmol/g, 0.40 mM). After assembling the sequence, the Dde protecting group at the lysine α-amino group was removed by agitating with a solution of hydrazine monohydrate in DMF (2% *v*/*v*, 3 × 25 mL, 10 min). Then, the resin was washed with DMF (1 min × 2) and DCM (1 min × 2). The peptide was cleaved from the peptidyl resin using a mixture of acetic acid/trifluoroethanol (TFE)/DCM (1:2:7, *v*/*v*/*v*) by stirring for 2 h at room temperature to afford the linear peptide with protected side chains. The peptide was collected by filtration and washed with DCM (3 × 15 mL). The filtrate containing the peptide was evaporated under reduced pressure. To remove acetic acid from the cleaved peptide, the peptide was re-dissolved in 25 mL of DCM and precipitated with 50 mL of hexane, and the process was repeated four times until the peptide was completely dried and acetic acid was completely removed. The protected linear peptide was obtained as a solid white powder that was dried under vacuum overnight. The cyclization of the linear peptide was carried out by dissolving the peptide in DMF/DCM mixture (250 mL, 4:1 *v*/*v*). Then HOAt (223 mg, 1.64 mmol, 4 equiv) and DIC (290 μL, 1.86 mmol, 4.5 equiv) were added to the peptide solution. The mixture was stirred at room temperature for 12 h. The completion of the cyclization was confirmed with mass checked by MALDI-TOF(shown in the Supplementary Materials). After the reaction was completed, the solvents were removed under reduced pressure using a rotary evaporator. The cleavage of the protecting groups from the amino acid side chains was carried out by adding reagent “R” containing trifluoroacetic acid (TFA)/thioanisole/ethanedithiol(EDT)/anisole (90:5:3:2, *v*/*v*/*v*/*v*, 25 mL) to the cyclic peptide. The mixture was shaken at room temperature for 6 h, and cold ether was added to precipitate the crude peptide. The peptide was collected and purified by reversed-phase Hitachi HPLC (L-2455) using a C18 column (Waters XBridgeTM BEH130 Prep C18 OBDTM 10 μm ODS reversed-phase column) (2.1 × 25 cm) using a gradient system of acetonitrile/water containing 0.1% TFA to yield (~40%) the cyclic peptide [W(WR)_4_K(*β*Ala)]. MALDI-TOF (*m/z*) [C_88_H_115_N_29_O_11_]: calcd 1753.9331; found 1755.0300 [M + 2H]^+^, 1792.9736 [M + K]^+^ (shown in Supplementary Materials).

#### 3.2.2. Synthesis of [(WR)_4_WK(*β*Ala-Thiopropionic acid)] (P2) and [(WR)_4_WK(*β*Ala-Thiopropionyl-ethaneamine] (P3)

[W(WR)_4_K(*β*Ala)] peptide (26.30 mg, 0.015 mmol) was dissolved in 1 mL of anhydrous DMF, then a solution of 3-(tritylthio)propionic acid (6.1 mg, 0.0175 mmol, 1.16 equiv) dissolved in 1 mL of DMF containing DIPEA (15.7 µL, 6 equiv), and HBTU (6.6 mg, 0.0175 mmol) were added to the peptide solution, and the stirring was continued for 6 h under N_2_ at room temperature. After the reaction was completed, the solvent was evaporated under reduced pressure until complete dryness. The dry solid peptide was treated by a mixture of TFA/triisopropyl silane (TIS)/H_2_O (10 mL, 7:2:1, *v*/*v*/*v*) and stirred at room temperature for 4 h to cleave the trityl group. Then the peptide was precipitated with cold diethyl ether and centrifuged, to yield the cyclic peptide [(WR)_4_WK(*β*Ala-thiopropionic acid)] **(P2)** (23.3 mg, 93% yield): MALDI-TOF (*m/z*) [C_91_H_119_N_29_O_12_S]: calcd 1841.9314; found 1842.8826 [M + H]^+^, 1864.8585 [M + Na] (shown in Supplementary Materials).

The peptide [(WR)_4_WK(*β*Ala-thiopropionic acid)] **P2** (20 mg, 0.011 mmol) containing free thiol group was further functionalized by reaction with 2-(pyridinyldithio)-ethaneamine hydrochloride (7.35 mg, 0.033 mmol, 3 equiv) prepared using cystamine and dithiopyridine according to the method described by Zugates et al. [37] in methanol containing a catalytic amount of acetic acid. The reaction was stirred overnight at room temperature, and then the peptide **(P3)** was collected by precipitation with cold ether and purified by reversed-phase HPLC as mentioned above. The yield was 87.75% (17.55 mg). MALDI-TOF (*m/z*) [C_93_H_124_N_30_O_12_S_2_]: calcd 1916.9456; found 1917.9089 [M + H]^+^, 1941.9098 [M + Na]^+^ (shown in Supplementary Materials).

#### 3.2.3. Synthesis of Paclitaxel-2-*O*-Hemiglutarate

Paclitaxel-2-*O*-hemiglutarate was prepared according to the method described by Sundaram et al. [38]. In brief, paclitaxel (100 mg, 0.117 mM) and glutaric anhydride (13.70 mg, 0.12 mM) were dissolved in 15 mL of DCM, and 10 µL of dry pyridine was added to the reaction mixture as a base catalyst. The reaction was stirred under nitrogen for 48 h at room temperature. The progress of the reaction was monitored by TLC using hexane:ethyl acetate (7:3, *v*/*v*). The crude mixture was purified by silica gel chromatography. The compound was obtained as a white solid (76 mg, 95% yield). HR-MS (ESI) (*m/z*) [C_52_H_57_NO_17_]: calcd 967.3626; found 968.3479 [M + H]^+^.

#### 3.2.4. General Procedure for the Synthesis of PTX-1 from Coupling of [W(WR)_4_K(βAla)] Peptide with PTX-2-*O*-Hemiglutarate

PTX-2-*O*-hemiglutarate (1.2 equiv, 11.6 mg, 1.2 mM), benzotriazol-1-yloxytripyrrolidino- phosphoniumhexafluorophosphate (PyBOP, 1.5 equiv, 7.81 mg, 0.015 mM), and 1-hydroxybenzo- triazole (HOBt, 2.5 equiv, 3.38 mg, 0.025 mM) were mixed together in a glass vial under nitrogen atmosphere using 2 mL of anhydrous DMF to activate the carboxylate group of the hemiglutarate moiety for 10 min. Then a solution of [W(WR)_4_K(*β*Ala)] peptide (1 equiv, 17.54 mg, 1 mM) in DMF containing DIPEA (3 equiv, 5 µL, 0.03 mM) was added to the activated PTX-2-*O*-hemiglutarate mixture dropwise over 10 min. The reaction mixture was stirred for 2–3 h in the dark under N_2_ gas. The progress of conjugation was monitored by checking the mass of a small sample taken from the reaction mixture by MALDI-TOF to confirm the formation of the PTX-peptide conjugate. After the reaction was completed, the crude product was precipitated by adding cold diethyl ether and centrifuged. The peptide was dried under nitrogen gas, dissolved in acetonitrile/water (50% *v*/*v*), and purified by RP-HPLC affording [W(WR)_4_K(*β*Ala-hemiglutarate-2-*O*-PTX)] conjugate (**PTX-1**), which was lyophilized (5.5 mg, 18.9% yield). MALDI-TOF (*m/z*) [C_140_H_170_N_30_O_27_]: calcd 2703.2852; found 2703.9563 [M]^+^ (shown in Supplementary Materials).

#### 3.2.5. Synthesis of Camptothecin-20-*O*-Chloroformate

Camptothecin-20-*O*-chloroformate was prepared as described by Henne et al. [39]. In brief, CPT (863 mg, 2.5 mM) and DMAP (763 mg, 6.25 mM) were dissolved in 15 mL of dry CH_2_Cl_2_ under N_2_ atmosphere. Triphosgene (0.250 mg, 0.84 mM) dissolved in 5 mL of dry DCM was added to CPT solution at 0 °C. The mixture was stirred for a further 60 min. Then the reaction mixture was dissolved in 50 mL of DCM and extracted by addition of 1.0 M HCl solution (2 × 50 mL), and brine (2 × 50 mL). The organic layer was collected and dried over anhydrous Na_2_SO_4_. DCM was evaporated and used without further purification to give a pale-yellow powder (761 mg, 88% yield).

#### 3.2.6. Synthesis of CPT1 from Coupling of [W(WR)_4_K(βAla)] Peptide with CPT-20-*O*-Chloroformate

[W(WR)_4_K(*β*Ala)] (1 equiv, 17.54 mg, 1 mM) peptide was dissolved in 1 mL of anhydrous DMF, and CPT-20-*O*-chloroformate (1.2 equiv, 4.92 mg, 1.2 mM) dissolved in 5 mL of anhydrous DCM containing (3 equiv, 5 µL, 0.03 mM) of DIPEA was added to the peptide solution, and the reaction was continued for 24 h. The progress of conjugation was monitored by checking the mass using MALDI-TOF. Once the reaction was completed, the crude product was precipitated by adding cold diethyl ether and centrifuged, dried under N_2_. The dried solid compound was dissolved in acetonitrile/water (50% *v*/*v*) and purified by RP-HPLC. The purified CPT-20-*O*-formyl-[W(WR)_4_K(*β*Ala)] **(CPT1)** conjugate was lyophilized (7.56 mg, 33.40% yield). MALDI-TOF (*m/z*) [C_109_H_129_N_31_O_16_]: calcd 2128.0234; found 2128.6907 [M]^+^ (shown in Supplementary Materials).

#### 3.2.7. Synthesis of CPT2 from Coupling of [(WR)_4_WK(βAla-thiopropionyl-*S*-*S*-ethaneamine)] with CPT-20-*O*-chloroformate

CPT-20-*O*-chloroformate (1.2 equiv, 4.92 mg, 1.2 mM) was dissolved in 5 mL of anhydrous DCM containing DIPEA (3 equiv, 5 µL, 0.03 mM). The peptide (1 equiv, 17.54 mg, 0.1 mM) was dissolved in 1 mL of anhydrous DMSO and added dropwise over 10 min to CPT-20-*O*-chloroformate solution. The reaction mixture was stirred for 24 h at room temperature under N_2_, and the reaction progress was monitored using MALDI-TOF to check the formation of CPT-peptide conjugate. Once the reaction was completed, the crude product was precipitated by adding cold diethyl ether, centrifuged, and dried under N_2_. The dried solid compound was dissolved in acetonitrile/water (50%, *v*/*v*) and purified by RP-HPLC (6.21 mg, 25.8% yield). The purified [W(WR)_4_K(*β*Ala—formyl-*O*-20-CPT)] **(CPT2)** conjugate was lyophilized. MALDI-TOF (*m/z*) [C_114_H_138_N_32_O_17_S_2_]: calcd 2291.0359; found 2291.9724 [M]^+^ (shown in Supplementary Materials).

#### 3.2.8. Cell Culture

Human breast cancer cell line (MCF-7, ATCC# HTB-22™) cells and human embryonic kidney cells (HEK-293, ATCC# CRL-1573) were purchased from the American Type Culture Collection (Manassas, VA, USA). All MCF-7 cells were cultured using 75 cm^2^ cell culture flasks. A complete Eagle’s minimum essential medium (EMEM) (ATCC^®^ 30-2003^™^) containing fetal bovine serum (FBS, 10%), and penicillin-streptomycin solution (1%, penicillin (10,000 units) and streptomycin (10 mg in 0.9% NaCl) were used under the atmosphere of CO_2_ (5%) and air (95%) at 37 °C.

#### 3.2.9. Antiproliferative Assay

The comparative antiproliferative assay was conducted using the MTS cell viability method to evaluate the potency of the conjugates to inhibit the proliferation of MCF-7 breast cancer cells and HEK-293 normal embryonic kidney cells. The cells were seeded (5000/0.1 mL) in each well using a 96-well plate. The MCF-7 cells were treated with PTX and CPT drugs and their corresponding conjugates in the medium. The final concentrations of all the compounds were adjusted to be 5 μM. Cells were incubated with the treatments for 4 h. Then, the treatments were removed and replaced by the fresh medium and incubated for a further 72 h. A similar treatment was followed for the HEK-293 cell line with PTX1, PTX, and cyclic peptide [W(WR)_4_K(*β*Ala)] at 5 μM. The CellTiter 96 aqueous solution (Promega, Madison, WI, USA) was used to measure the cell viability based on the fluorescence intensity of them at 490 nm. Here, SpectraMax M2 microplate spectrophotometer was employed for the assay. The cell viability was calculated relatively based on the cell survival as [(OD value of cells treated with the test mixture of compounds) − (OD value of culture medium)]/[(OD value of control cells) − (OD value of culture medium)] × 100%.

## 4. Conclusion

A cyclic cell-penetrating peptide [W(WR)_4_K(*β*Ala)] was synthesized and used as a molecular cargo for hydrophobic anticancer drugs CPT and PTX. The drugs, CPT and PTX, were functionalized with a linker and conjugated with the peptide. The conjugates showed better water solubility due to their attachment to [W(WR)_4_K(*β*Ala)] which promises improved bioavailability and pharmacokinetic profiles. The antiproliferative activities of the peptide-drug conjugates were less than the free hydrophobic drugs in MCF-7 after 72 h incubation, which suggests the prodrug formation of CPT and PTX. The CPT conjugate named [W(WR)_4_K-(*β*Ala)]-*S*-*S*-CPT (CPT2) was found to be more cytotoxic when compared with [W(WR)_4_K-(*β*Ala)]-CPT (CPT1), suggesting the fast release of CPT analog in this conjugate. Future studies will be conducted to explore the potency of these conjugates on different cell lines and to understand the actual mechanism for the release of PTX and CPT from the peptide and improving the conjugation strategy of the drugs to the peptide.

## Supplementary Materials

The MALDI spectra of selected compounds are provided.
