# Supplementary material for: Synthesis and Antiproliferative Activities of Conjugates of Paclitaxel and Camptothecin with a Cyclic Cell-Penetrating Peptide
Source: Molecules. 2019 Apr 11;24(7):1427. doi: 10.3390/molecules24071427 (PMC6480016; doi:10.3390/molecules24071427)
Supplement: Supplementary file 1 [file molecules-24-01427-s001.pdf]

Supplementary information

Naglaa S. El-Sayed[1,2], Amir N. Shirazi[1,3], Muhammad Imran Sajid[1,4], Shang Eun Park[1], Keykavous Parang[1,*] and Rakesh K. Tiwari[1,*]

1 Center for Targeted Drug Delivery, Department of Biomedical and Pharmaceutical Sciences, Chapman University School of Pharmacy, Harry and Diane Rinker Health Science Campus, Irvine, California 92618, United States; nibrahim@chapman.edu (N.S.EL-Sayed); ashirazi@ketchum.edu (A.N.S.); park327@mail.chapman.edu(S.E.P.); sajid@chapman.edu (M.I.S.); parang@chapman.edu (K.P.); tiwari@chapman.edu (R.K.T.)

2 Cellulose and Paper Department, National Research Center, Dokki 12622, Cairo, Egypt

3 Department of Pharmaceutical Sciences, College of Pharmacy, Marshall B. Ketchum University, Fullerton, CA 92831, USA

4 Faculty of Pharmacy, University of Central Punjab, Lahore, Pakistan

* Correspondence: parang@chapman.edu; Tel.:+1-714-516-+5489 (K.P.); tiwari@chapman.edu; Tel.: +1-714-516-5483 (R.K.T.)

Mass spectroscopy data of selected synthesized compounds:

P1

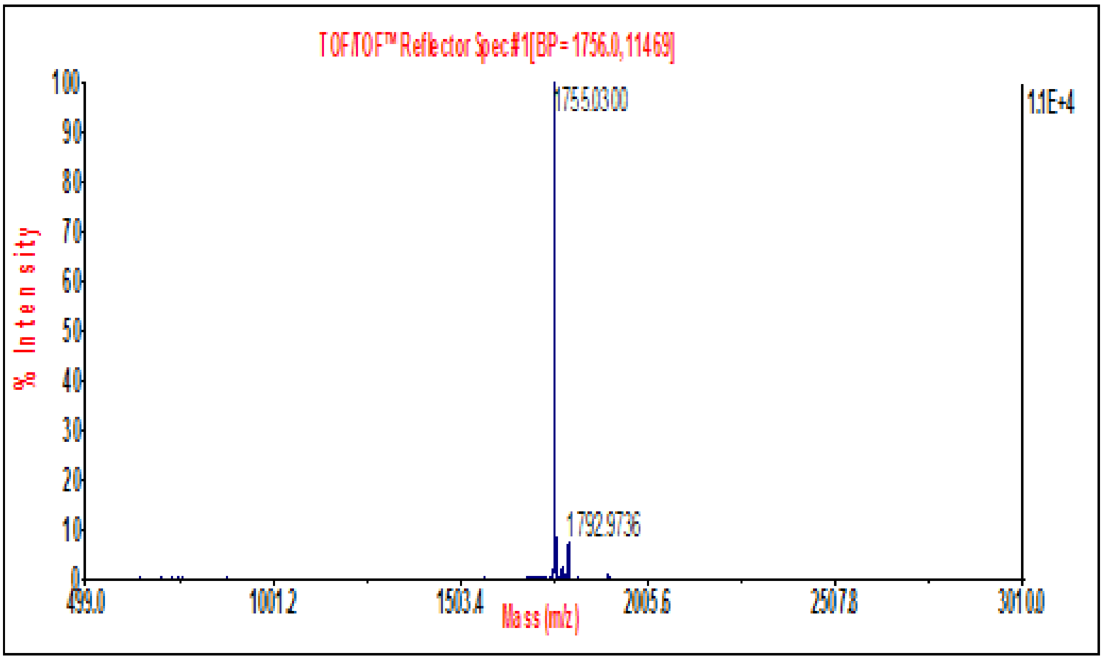

P2

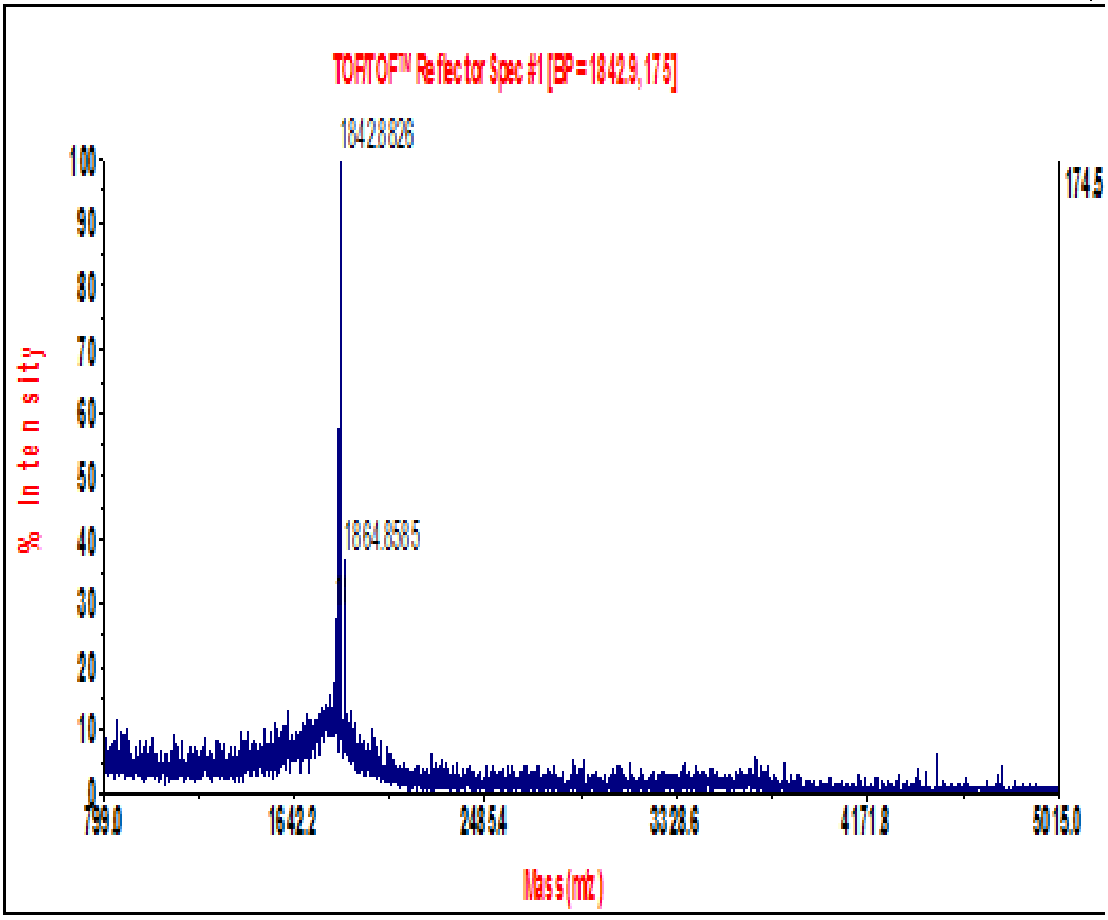

TOFTOF™ Reflector Spec #1 [BP = 1842.9, 175]
1842.8826
1864.8585
174.5
% Intensity
Mass (m/z)
799.0
1642.2
2485.4
3328.6
4171.8
5015.0

P3

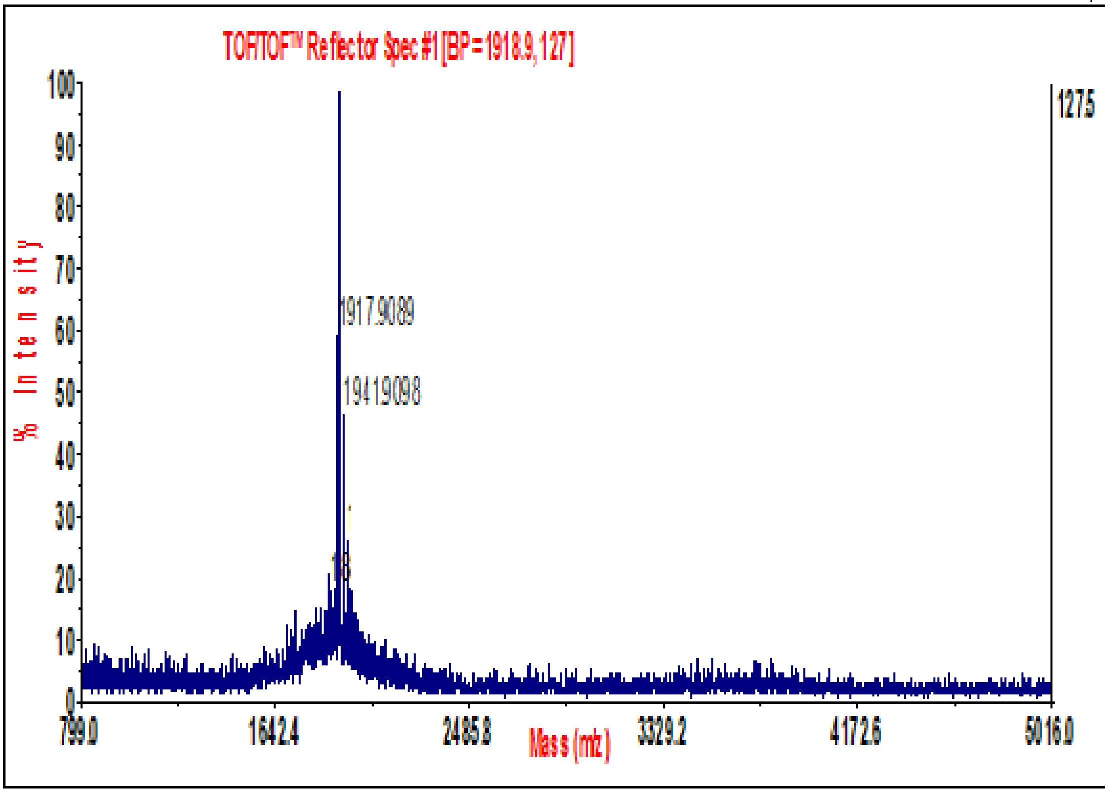
TOF/TOF™ Reflector Spec #1 [BP=1918.9, 127]
% Intensity
Mass (m/z)
127.5
1917.9089
1941.9098
100
90
80
70
60
50
40
30
20
10
0
799.0
1642.4
2485.8
3329.2
4172.6
5016.0

PTX1

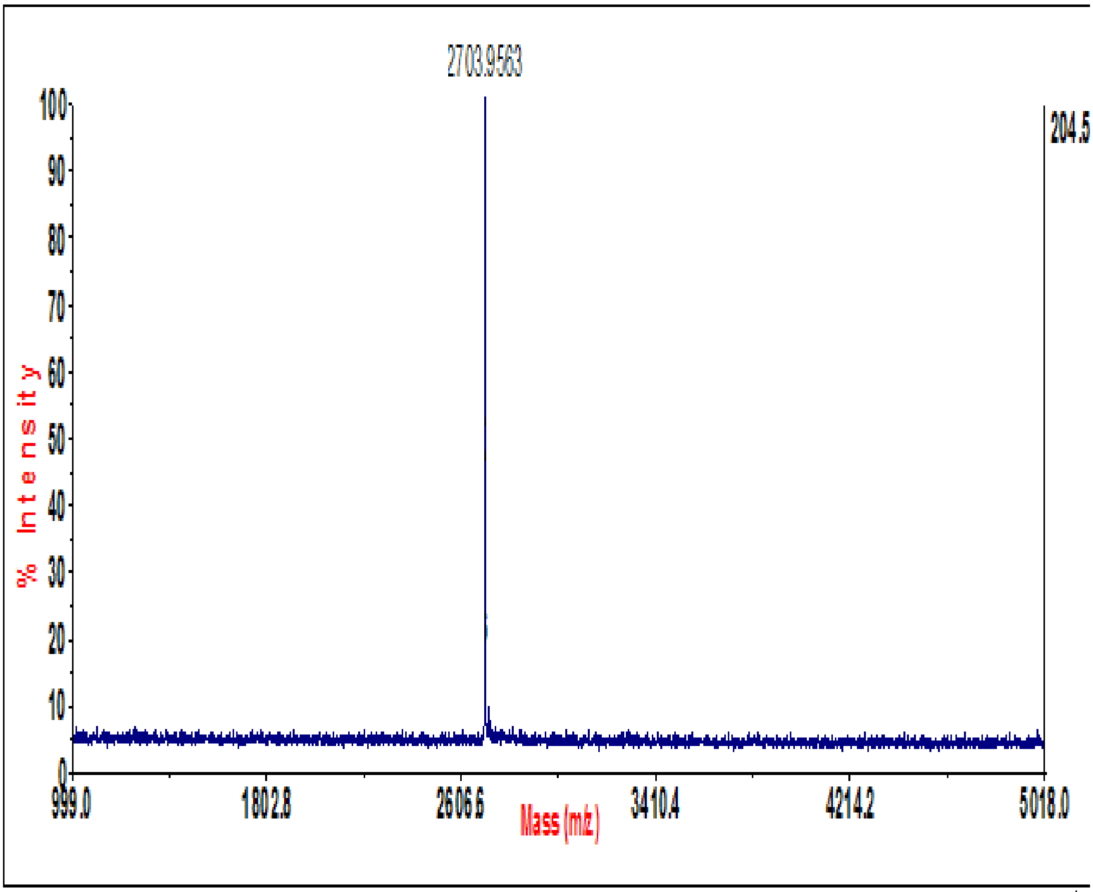

2703.9563
204.5
100
90
80
70
60
50
40
30
20
10
0
% Intensity
999.0
1802.8
2606.6
Mass (m/z)
3410.4
4214.2
5018.0

CPT1

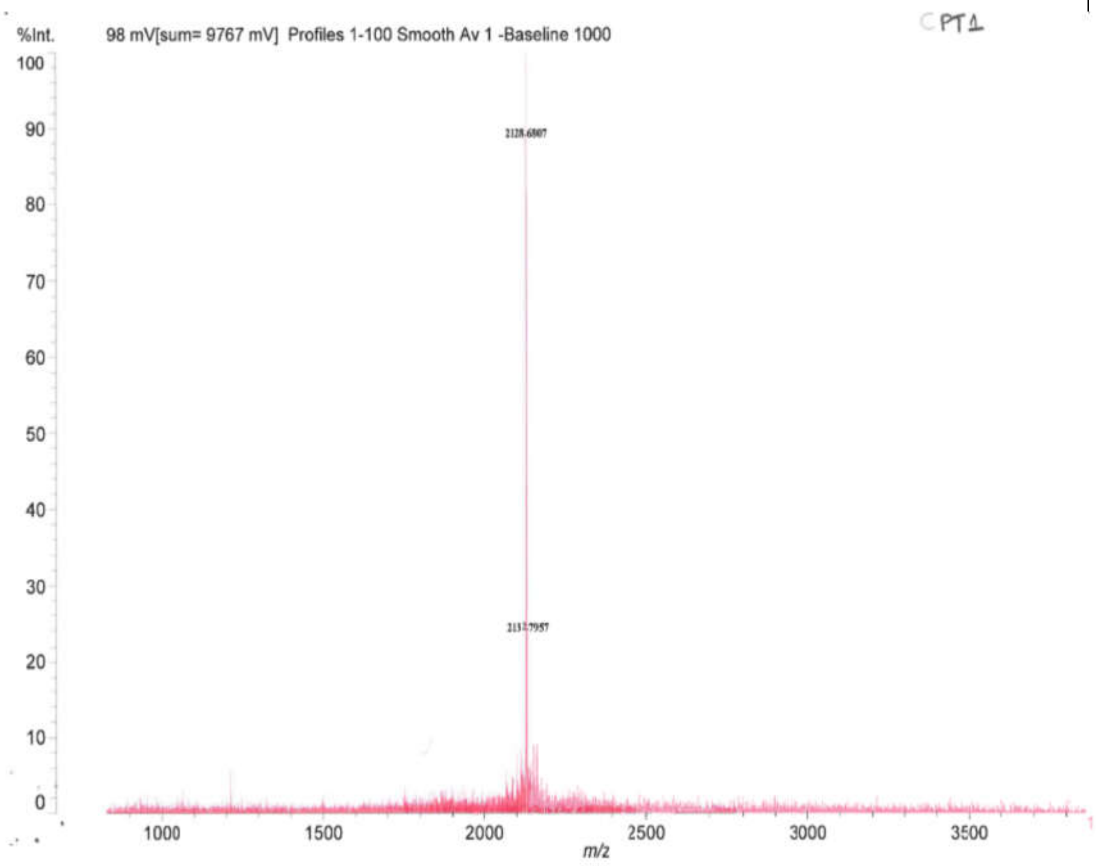

%Int.
98 mV[sum= 9767 mV] Profiles 1-100 Smooth Av 1 -Baseline 1000
CPT1
m/z

CPT2

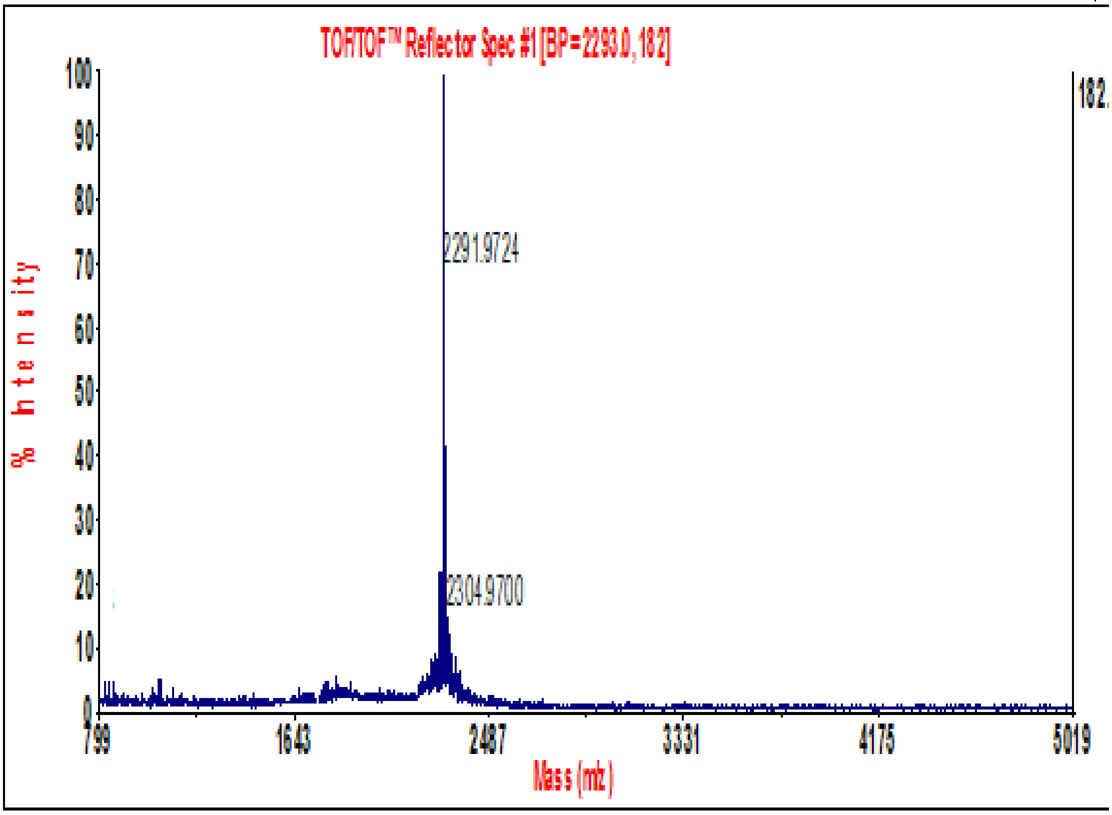

TOFTOF™ Reflector Spec #1 [BP=2293.0, 182]
2291.9724
2304.9700
% Intensity
Mass (m/z)
